# Decursin inhibits retinal neovascularization via suppression of VEGFR-2 activation

**Published:** 2009-09-12

**Authors:** Jeong Hun Kim, Jin Hyoung Kim, You Mie Lee, Eun-Mi Ahn, Kyu-Won Kim, Young Suk Yu

**Affiliations:** 1 Fight against Angiogenesis-Related Blindness (FARB) Laboratory, Department of Ophthalmology, College of Medicine, Seoul National University and Seoul Artificial Eye Center Clinical Research Institute, Seoul National University Hospital, Seoul, Korea; 2 Department of Natural Sciences, School of Life Sciences and Biotechnology, Kyungpook National University, Daegu, Korea; 3 Department of Herbal Foodceutical Science, Daegu Hanny University, Daegu, Korea; 4 Neurovascular Coordination Research Center, College of Pharmacy and Research Institute of Pharmaceutical Sciences, Seoul National University, Seoul, Korea

## Abstract

**Purpose:**

Pathologic angiogenesis in the retina leads to the catastrophic loss of vision. Retinopathy of prematurity (ROP), a vasoproliferative retinopathy, is a leading cause of blindness in children. We evaluated the inhibitory effect of decursin on retinal neovascularization.

**Methods:**

Anti-angiogenic activity of decursin was evaluated by vascular endothelial growth factor (VEGF)-induced proliferation, migration, and in vitro tube formation assay of human retinal microvascular endothelial cells (HRMECs). We also used western blot analysis to assess inhibition of vascular endothelial growth factor receptor-2 (VEGFR-2) phosphorylation by decursin. After intravitreal injection of decursin in a mouse model of ROP, retinal neovascularization was examined by fluorescence angiography and vessel counting in cross-sections. The toxicity of decursin was evaluated through 3-(4,5-dimethylthiazol-2-yl)-2,5-diphenyltetrazolium bromide (MTT) assay in HRMECs as well as histologic and immunohistochemistry examination for glial fibrillary acidic protein in the retina.

**Results:**

Decursin significantly inhibited VEGF-induced proliferation, migration, and the formation of capillary-like networks of retinal endothelial cells in a dose-dependent manner. Decursin inhibited VEGF-induced phosphorylation of VEGFR-2, blocking the VEGFR-2 signaling pathway. When intravitreously injected, decursin dramatically suppressed retinal neovascularization in a mouse model of ROP. Even in a high concentration, decursin never induced any structural or inflammatory changes to cells in retinal or vitreous layers. Moreover, the upregulation of glial fibrillary acidic protein expression was not detected in Mueller cells.

**Conclusions:**

Our data suggest that decursin may be a potent anti-angiogenic agent targeting the VEGFR-2 signaling pathway, which significantly inhibits retinal neovascularization without retinal toxicity and may be applicable in various other vasoproliferative retinopathies as well.

## Introduction

Angiogenesis plays a central role in tissue development and repair. A balance of many stimulating or inhibiting factors tightly regulate these processes [1]. However, when that balance is disrupted, stimulation with angiogenic factors, such as vascular endothelial growth factor (VEGF) and fibroblast growth factor (FGF), allows vascular endothelial cells to proliferate and migrate into the surrounding tissue. These newly formed, dysfunctional blood vessels are leaky, fragile and prone to rupture, and hemorrhagic, a condition that is associated with fibrous proliferation [2]. Therefore, pathologic angiogenesis in the retina leads to retinal edema, retinal or vitreous hemorrhage, and finally tractional retinal detachment, which can result in catastrophic loss of vision [3]. Pathologic angiogenesis is the major cause of vision loss at all ages, including retinopathy of prematurity (ROP) in children, diabetic retinopathy (DR) in young adults, and age-related macular degeneration (AMD) in the elderly [4].

ROP is a leading cause of blindness in children [5]. Although the cellular and molecular processes remain incompletely understood, ROP is known to be a vasoproliferative retinopathy in premature infants that occurs through vaso-obliteration followed by pathologic angiogenesis in developing retinal vasculature [6]. Therefore, oxygen-induced retinopathy (OIR) in a mouse model, which reflects the current understanding of the pathogenesis of the disease, is based on hyperoxia-induced vaso-obliteration of capillaries in mouse pups and their subsequent return to room air. This triggers retinal angiogenesis, starting from the inner retina and characterized by growing into the vitreous [7].

In ROP, retinal neovascularization followed by vaso-obliteration appears to be driven by relative tissue hypoxia. Increased VEGF production in response to hypoxia leads to pathologic retinal angiogenesis. VEGF and the VEGFR system are known to be the main regulators of angiogenesis, in which VEGF interacts with the high-affinity tyrosine kinase receptors VEGFR-1 and VEGFR-2 [8]. In particular, VEGFR-2 signaling is essential not only for vascular endothelial proliferation but also for cell migration or morphogenesis, including tube formation. For angiogenesis, VEGFR-2 efficiently activates the phospholipase-Cγ and protein kinase C pathways, and its downstream *c-Raf*-MEK-MAP kinase pathway, which is mainly regulated by a single autophosphorylation of 1175-tyrosine [9,10].

*Angelica gigas* Nakai has been traditionally known as a medicinal plant in East Asia. Decursin, isolated from the root of this plant [11], has been reported to have variable pharmacologic qualities, such as neuroprotection [12], antibacterial properties [13], and anticancer activities [14,15]. In the course of our research regarding new angiogenesis inhibitors from natural products, we recently found decursin to be a potent angiogenesis inhibitor: It effectively inhibited tumor angiogenesis as well as VEGF-induced angiogenic processes in vitro and in vivo, including proliferation, migration, and tube formation of human umbilical-vein endothelial cells and neovascularization in chick chorioallantoic membrane [16]. In addition, we demonstrated that decursin inhibits VEGF-induced phosphorylation of VEGFR-2 and its signaling pathway [16].

In our study, we showed that decursin significantly inhibits retinal neovascularization via suppression of VEGFR-2 activation. Decursin significantly inhibited VEGF-induced proliferation of human retinal microvascular endothelial cells (HRMECs) in a dose-dependent manner, which could be related to suppression of VEGFR-2 phosphorylation and effectively inhibited VEGF-induced migration and tube formation of HRMECs. In addition, when decursin was intravitreally injected, retinal neovascularization in OIR was significantly suppressed. Interestingly, in quantities of up to 50 μM, which is five times the effective therapeutic concentration [16], decursin never affected the viability of HRMECs. Moreover, decursin induced neither the activation of Mueller cells, which are thought to play an important role both structurally and functionally in the retina [17], nor any structural change.

## Methods

### Extraction of decursin

The roots (Professor Eun-Mi Ahn, Daegu Hanny University, Daegu, Korea) of *Angelica gigas* Nakai (Umbelliferae family) were extracted serially with methanol, ethyl acetate, and N-butanol, and fractionated. From the ethyl acetate fraction, decursin was isolated using silica-gel column chromatography. After column chromatography, the structure of purified decursin was characterized by gas chromatography (GC; Shimadzu, Kyoto, Japan), nuclear magnetic resonance (JEOL JNM-LA 400; Jeol Ltd., Tokyo, Japan), and mass spectroscopy (JEOL-AX 505WA; Jeol Ltd.). As our previous report [16], decursin was isolated from the ethylacetate fraction using silica gel column chromatography. After column chromatography, decursin (C19H20O5) with a molecular weight of 328 was characterized.

### Animals

Eight weeks old C57BL/6 mice were kept in standard 12 h dark-light cycles and approximately 23 °C room temperature. C57BL/6 mice were purchased from Samtako (Daejeon, Korea). Care, use, and treatment of all animals in this study were in strict agreement with the Association for Research in Vision and Ophthalmology Statement for the Use of Animals in Ophthalmic and Vision Research.

### Cell culture

HRMECs were purchased from Cell systems (Kirkland, WA) and grown on attachment factor–coated plates in complete medium (Cell Systems, Kirkland, WA) or in M199 medium supplemented with 20% fetal bovine serum, 3 ng/ml basic FGF (Millipore, Bedford, MA), and 10 U/ml heparin (Sigma, St. Louis, MO). HRMECs used in this study were taken from passages four to six.

### Proliferation assay with [^3^H]-thymidine

HRMECs were seeded in gelatin-coated 48-well plates at 1×10^4^ cells per well. Cells were treated with 20 ng/ml VEGF or 1 to 50 μM decursin. After 24 h, 0.5 µCi per well of [^3^H]-thymidine was added, and the plates were incubated for 16 h. Cells were fixed with methanol, washed with distilled water, treated with 5% trichloroacetic acid, and solubilized with 0.3 N sodium hydroxide. HRMECs were fixed with two 20 min applications of 80% methanol and treated with two 10-min application 5% trichloroacetic acid. Cell-associated radioactivity was determined by liquid scintillation counter (Perkin Elmer, Waltham, MA).

### Western blot analysis

HRMECs were seeded in 100-mm dishes (5×10^5^ cells) and were incubated for 12 h in either 5 μM decursin or 20 ng/ml VEGF. The cell lysates were separated by 12.5% sodium dodecyl sulfate PAGE, followed by transfer to polyvinylidene fluoride membranes (Millipore) using standard electroblotting procedures. Blots were then blocked and immunolabeled overnight at 4 °C with the primary antibodies, anti-VEGFR-2, antiphosphorylated VEGFR-2 (Cell Signaling Technology, Beverly, MA) and anti-β-actin (Upstate Biotechnology, Lake Placid, NY). Immunolabeling was detected with an enhanced chemiluminescence kit (Amersham Life Science, Buckinghamshire, UK), using the manufacturer’s instructions.

### Scratch wound migration assay on retinal endothelial cells

Cell migration was evaluated with scratch wound migration assay modified from our previous description [18]. HRMECs (1×10^5^ cells) were plated onto gelatin-coated culture dishes at 90% confluence and were wounded with a razor blade. Wound was made by scraping across with the razor blade. After the wounding, plates were rinsed with a serum-free medium. Then the wounded monolayers were incubated in 10 μM decursin or 20 ng/ml VEGF (Sigma) for 12 h. The cells were fixed with absolute methanol and stained with Giemsa solution (BDH Laboratory Supplies, London, UK). Cells were observed under a light microscope (BX51; Olympus, Tokyo, Japan) and photographed at a magnification of 200×. Migration was determined by counting the number of cells that moved beyond the reference line in randomly selected fields.

### Tube formation assay on retinal endothelial cells

Tube formation was assayed as per our previous description [19]. HRMECs (1×10^5^ cells) were inoculated on the surface of Matrigel basement membrane matrix (BD Biosciences, Franklin Lakes, NJ) and treated with 10 μM decursin or 20 ng/ml VEGF (Sigma) for 18 h. The morphologic changes of the cells and tubes formed were observed under a light microscope and photographed at a magnification of 200×. Tube formation was quantified by counting the number of connected cells in randomly selected fields at a magnification of 200× and dividing that number by the total number of cells in the same field.

### Oxygen-induced retinopathy

OIR was induced as described by Smith et al. [7], with some modifications [20-22]. Briefly, newborn mice were randomly assigned to experimental and control groups. At postnatal day (P) 7, five to seven pups in the experimental group were exposed to hyperoxia (75%±0.5% O_2_) for five days (P7–P11) and then returned to normoxia (room air) for five days. Neovascularization occurred on return to normoxia and peaked at P17. To assess the anti-angiogenic activity of decursin, we intravitreously injected the pups with 5 µM decursin in 1 µl phosphate-buffered saline on P14, when retinal neovascularization began. There were at least 10 animals in each group.

### Qualitative assessment of retinal neovascularization by fluorescence angiography

As we noted in our previous description [20-22], at P17, deeply anesthetized mice were perfused through the tail vein with fluorescein-conjugated dextran (molecular weight 500,000; Sigma) dissolved in phosphate-buffered saline (PBS; 137mM NaCl, 10mM phosphate, 2.7mM KCl, pH 7.4). After 1 h of perfusion, the eyes were enucleated and fixed in 4% paraformaldehyde for 2 h. The retinas were dissected, flat-mounted in Dako mounting medium (DakoCytomation, Glostrup, Denmark), and viewed by fluorescence microscopy (BX50; Olympus, Tokyo, Japan) at a magnification of 4×.

### Quantitative assessment of retinal neovascularization by counting vascular lumens

As we noted in our previous description [20-22], at P17, the eyes were removed, fixed in 4% paraformaldehyde for 24 h, and embedded in paraffin. Sagittal sections of 5 µm, each 30 µm apart, were cut through the cornea parallel to the optic nerve. The sections were stained with hematoxylin and eosin to allow assessment of retinal vasculature via light microscopy. Any vascular lumens on the vitreal side of the inner limiting membrane were counted in at least 10 sections from each eye by two independent observers blind to treatment (Jeong Hun Kim and Jin Hyoung Kim). The average number of intravitreal vessels per section was calculated for each group.

### Histologic examination and immunohistochemistry

We gave six 8-week-old C57BL/6J mice an intravitreal injection of 50 µM decursin, which is five times the effective therapeutic concentration. Seven days later, the mice were euthanized by CO_2_ by inhalation and their eyes were enucleated. Enucleated globes were fixed in 4% paraformaldehyde for 24 h and embedded in paraffin. Sagittal sections of 5 µm, each 30 µm apart, were cut through the cornea parallel to the optic nerve. The sections were stained with hematoxylin and eosin, and histologic examination was performed via light microscopy by magnification of 400×. For immunohistochemistry for glial fibrillary acidic protein (GFAP), sections were deparaffinized and hydrated by sequential immersion in xylene and graded alcohol solutions. Sections were treated with proteinase K for 5 min at 37 °C and then treated for 10 min with normal serum obtained from the same species in which the secondary antibody was developed to block nonspecific staining. Slides were incubated overnight at 4 °C with 1:100 anti-GFAP (Dako, San Francisco, CA), and 1:400 fluorescein isothiocyanate–conjugated immunoglobulin G (Santa Cruz Biotechnology, Santa Cruz, CA) was used as a secondary antibody. The slides were mounted and observed with fluorescence microscopy. The excitation and emission wavelengths were 495 nm and over 515 nm.

### 3-(4,5-dimethylthiazol-2-yl)-2,5-diphenyltetrazolium bromide assay

Cell viability was evaluated with 3-(4,5-dimethylthiazol-2-yl)-2,5-diphenyltetrazolium bromide (MTT) assay. HRMECs (1×10^5^ cells) were plated in 96 well plates and cultured overnight. Cells were treated with 1 to 100 µM decursin for 48 h. The medium was then replaced with fresh medium containing 0.5 mg/ml MTT, and the cells remained in place for 4 h. After incubation, the medium was carefully removed from the plate, and dimethyl sulfoxide was added to solubilize formazan produced from MTT by the viable cells. Absorbance was measured at 540 nm using a microplate reader (Molecular Devices, Sunnyvale, CA).

### Statistical analysis

Statistical differences between groups were evaluated with the Student’s unpaired *t*-test (two-tailed). Values provided here are means±SD. A p value ≤0.05 was considered significant.

## Results

### Decursin inhibits VEGF-induced proliferation of HRMECs by blocking VEGFR-2 phosphorylation

**Figure 1:**
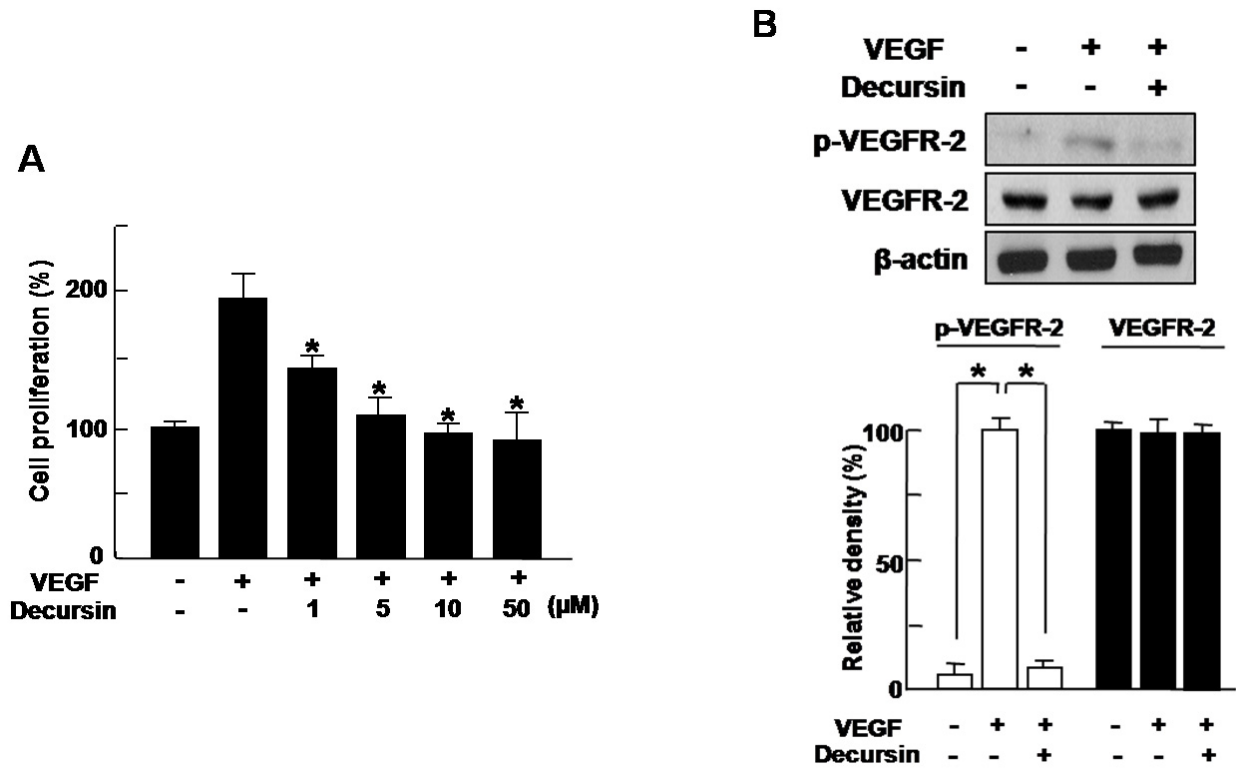
Decursin inhibits VEGF-induced migration and tube formation of HRMECs. **A**: Human retinal microvascular endothelial cells (HRMECs) were treated with 20 ng/ml vascular endothelial growth factor (VEGF) or 1–50 µM decursin for 24 h. A cell-proliferation assay with [^3^H]-thymidine was performed. Each value represents the mean (±SD) of three independent experiments. The asterisk indicates a p<0.05. **B**: HRMECs were treated with 20 ng/ml VEGF or 10 µM decursin for 5 min. Western blot analysis using phospho- (p-)VEGFR-2 and vascular endothelial growth factor receptor-2 (VEGFR-2) antibodies was performed, with β-actin serving as the loading control. Figures were selected as representative data from three independent experiments. Quantitative analysis was performed by measuring the intensity relative to the control. Each value represents means±SEM from three independent experiments. The asterisk indicates a p<0.05. The size of scale bars in figure **A, B** were 100 µm.

To investigate the effect of decursin on VEGF-induced proliferation of retinal endothelial cells, we used 1–50 µM concentrations of decursin on HRMECs. With the treatment of VEGF, proliferation of HRMECs increased 1.98 fold compared with control, which was significantly inhibited by cotreatment with decursin in a dose-dependent manner (Figure 1A).

We next addressed how decursin could block VEGF-induced proliferation of HRMECs. Because we recently found that decursin inhibits proliferation of endothelial cells induced by VEGF via inhibition of the VEGFR-2 signaling pathway [16], we investigated the effect of decursin on VEGFR-2 phosphorylation. At 5 min after VEGF treatment, VEGF-induced phosphorylation of VEGFR-2 was significantly increased; however, it was effectively blocked by cotreatment with decursin (Figure 1B).

### Decursin inhibits VEGF-induced migration and tube formation of HRMECs

**Figure 2:**
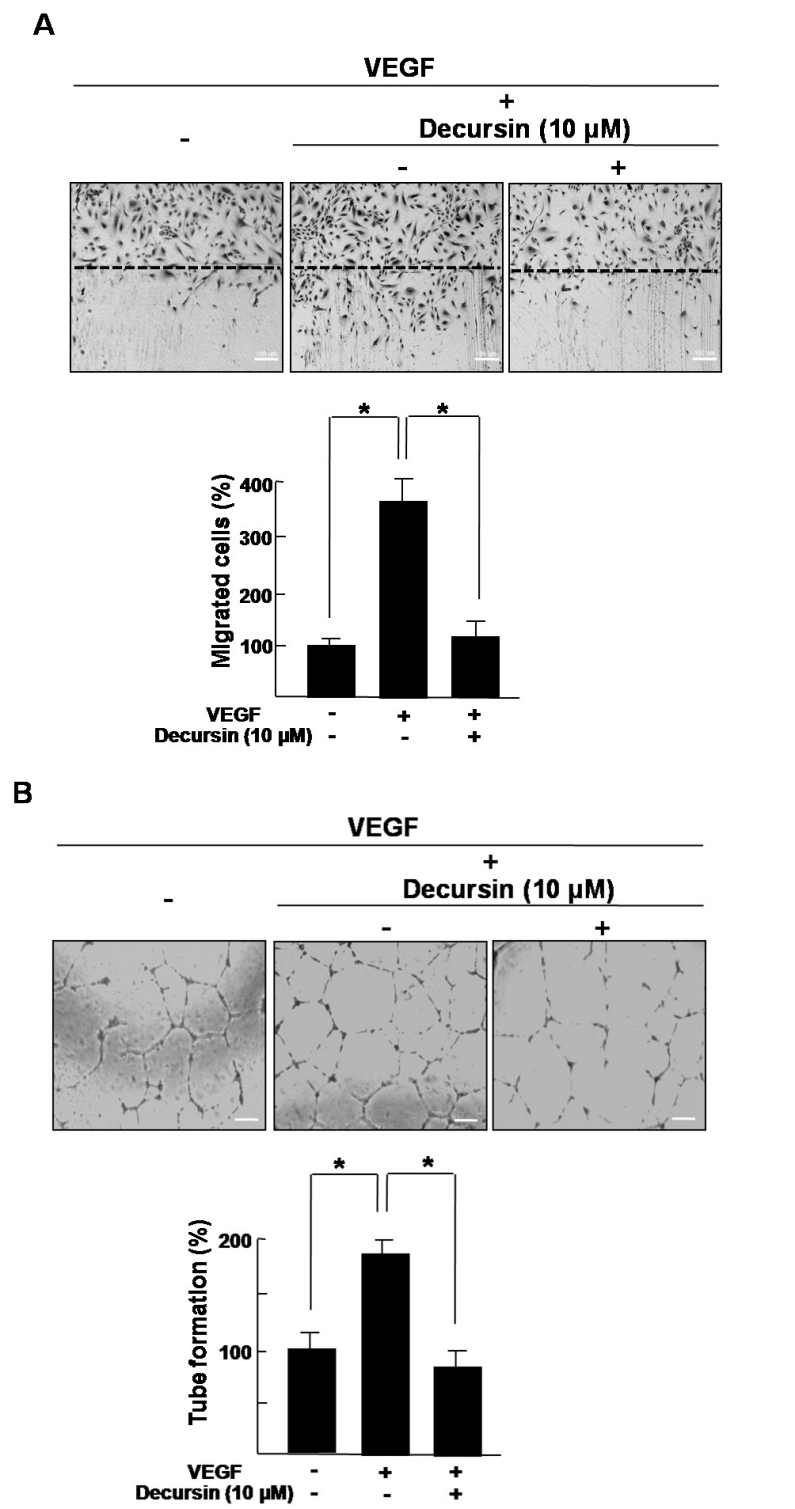
Decursin inhibits VEGF-induced migration and tube formation of HRMECs. **A**: Human retinal microvascular endothelial cells (HRMECs) were plated onto gelatin-coated culture dishes and wounded with a razor blade. Wound was made by scraping across with the razor blade. The wounded monolayers were treated with incubation in 10 µM decursin or 20 ng/ml vascular endothelial growth factor (VEGF) for 12 h. Figures were selected as representative data from three independent experiments. Migration was quantified by counting the number of cells that moved beyond the reference line. The basal migration of HRMECs that were left without decursin and VEGF were normalized to 100%. Each value represents the mean (±SD) of three independent experiments. The asterisk indicates a p<0.05. **B**: HRMECs were inoculated on the surface of the basement membrane matrix and treated with 10 µM decursin or 20 ng/ml VEGF for 18 h. Figures were selected as representative data from three independent experiments. The basal tube formation of HRMECs without decursin and VEGF was normalized to 100%. Each value represents the mean (±SD) of three independent experiments. The asterisk indicates a p<0.05.

To evaluate the anti-angiogenic activity of decursin on in vitro angiogenesis induced by VEGF, we performed a scratch wound assay for migration and tube formation assay of HRMECs. First, we addressed whether decursin could inhibit VEGF-induced migration. With the treatment of VEGF, the migration of HRMECs was enhanced 3.5 fold compared with the control, whereas the migratory ability was nearly eliminated by decursin, similar to the control (Figure 2A).

Next, to investigate the effect of decursin on the VEGF-induced maturation of migrated retinal endothelial cells, we evaluated tube network formation. In treatment with VEGF, formation of the capillary-like networks of HRMECs was extensive—a rate 1.8 fold that of control cells—but was completely inhibited by cotreatment with decursin (Figure 2B).

### Decursin inhibits retinal neovascularization in oxygen-induced retinopathy

**Figure 3:**
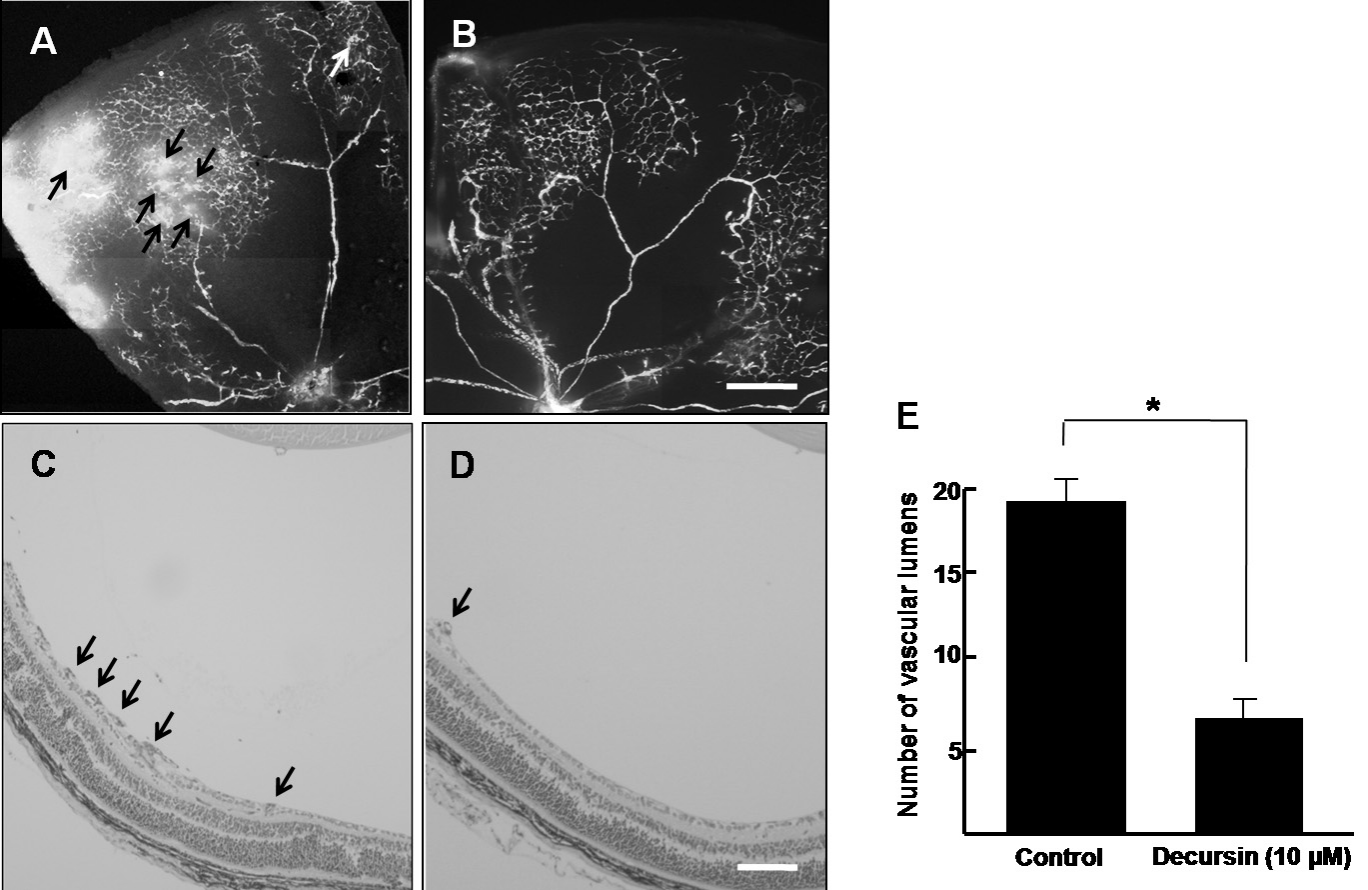
Decursin inhibits retinal neovascularization in oxygen-induced retinopathy. **A**, **B**: Retinal vasculature in control mice and decursin-treated mice with oxygen-induced retinopathy (OIR) was evaluated by fluorescence angiography using fluorescein-conjugated dextran. Whole-mount retinal preparation from postnatal day 17 (P17) control mice (**A**) and mice subjected to OIR and treated with 10 µM decursin (**B**) was performed after 1 h of perfusion with fluorescein-conjugated dextran. Arrows indicate neovascular tufts of intravitreous neovascularization. Figures were selected as representative data from three independent experiments with similar results. Scale bars equal 50 µm. Hematoxylin-stained cross-sections were prepared from P17 control mice **(C)** and mice subjected to OIR and treated with 10 μM of decursin **(D).** Arrows indicate the vascular lumens of new vessels growing into the vitreous. Figures were selected as representative data from three independent experiments with similar results. Scale bars equal 100 µm. **E**: Each value represents the mean (±SD) of three independent experiments. The asterisk indicates a p<0.05.

To investigate whether decursin could reduce retinal neovascularization, we injected 10 µM decursin intravitreously on P14 in OIR, and qualitatively and quantitatively analyzed the anti-angiogenic activity of decursin on retinal neovascularization. First, to investigate the anti-angiogenic activity of decursin on retinal neovascularization in OIR, we performed fluorescence angiography. On P17 in OIR, many neovascular tufts at the border of vascularized and nonvascularized retina were easily detected (Figure 3A), whereas they were significantly reduced in decursin-treated mice (Figure 3B).

Next, we quantitatively determined the anti-angiogenic activity of decursin on retinal neovascularization. As our previous description [20-22], vascular lumens on the vitreal side between the posterior capsule of the lens and the inner limiting membrane were counted. Compared with the lumens in control OIR mice (Figure 3C), the neovascular lumens in decursin-treated mice were dramatically decreased in the number of lumens (Figure 3D). Compared with neovascular lumens in control mice (19±1.5), those in decursin-treated mice were significantly reduced (6.5±1.2; p<0.05; Figure 3E).

### Decursin induced no cytotoxicity in retinal endothelial cells and no retinal toxicity

**Figure 4:**
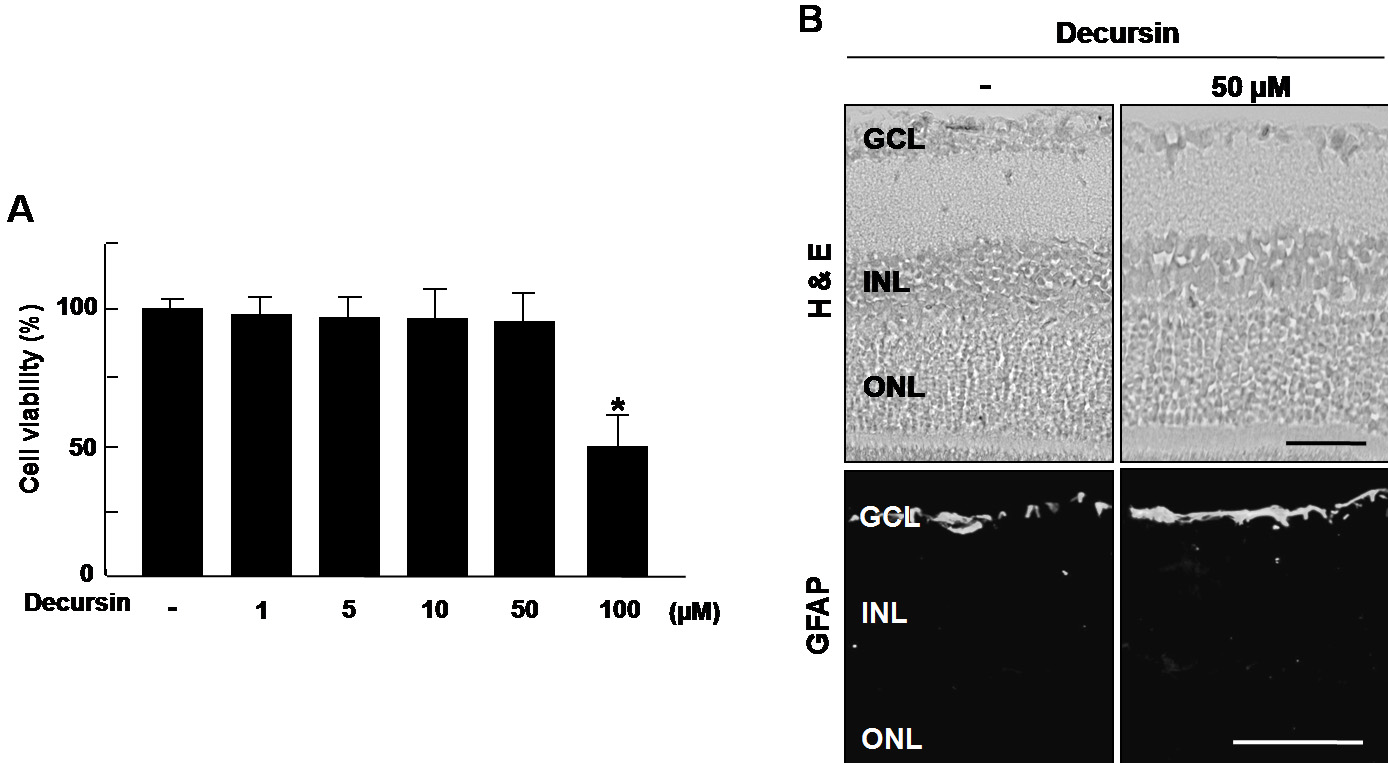
Decursin induced no cytotoxicity in retinal endothelial cells and no retinal toxicity. **A**: Human retinal microvascular endothelial cells were treated with 1–100 µM of decursin then incubated for 48 h. Cell viability was measured by 3-(4,5-dimethylthiazol-2-yl)-2,5-diphenyltetrazolium bromide (MTT) assay. Each value represents means±SEM from three independent experiments. The asterisk indicates a p<0.05. **B**: We injected intravitreously 50 µM decursin into mouse eyes, then enucleated the globes three days after treatment. Hematoxylin & eosin staining and immunohistochemistry for glial fibrillary acidic protein were performed. Figures were selected as representative data from three independent experiments with similar results. Scale bars equal 50 µm. Abbreviations: ganglion cell layer (GCL), hematoxylin and eosin (H&E), inner nuclear layer (INL), outer nuclear layer (ONL).

To determine whether decursin induces cytotoxicity in retinal endothelial cells, HRMEC viability was evaluated at doses ranging from 1 to 100 µM decursin. As shown in Figure 4A, no cytotoxicity was observed for decursin, even at amounts of up to 50 µM decursin, five times its therapeutic concentration [16].

To investigate the retinal toxicity of decursin, we performed histologic and immunohistochemistry studies for GFAP in the retina, following intravitreal injection of 50 µM of decursin. There were neither structural changes nor inflammatory cells in any retinal layers or in vitreous. Moreover, upregulation of GFAP expression was not detected in Müller cells, which are structurally and functionally critical in the retina (Figure 4B).

## Discussion

We demonstrated that decursin significantly inhibits retinal neovascularization in OIR without retinal toxicity. These results were supported by findings that decursin significantly inhibits VEGF-induced proliferation, migration, and tube formation of retinal endothelial cells. As supported by findings in our recent report, the anti-angiogenic activity of decursin in retinal neovascularization was closely related to inhibition of VEGFR-2 activation in retinal endothelial cells [16].

In the pathogenesis of retinal neovascularization in ROP, increased VEGF expression in the retina, followed by oxygen-induced vessel loss and subsequent hypoxia, leads to retinal neovascularization [6,7]. Therefore, VEGF plays a critical role in retinal neovascularization in ROP, which can be directly supported by data from previous reports showing that inhibition of VEGF significantly decreases retinal neovascularization in OIR [23,24]. In addition to the direct inhibition of VEGF, the regulation of hypoxia-inducible factor 1α (HIF-1α), a key transcriptional complex of angiogenic growth factors such as VEGF and platelet-derived growth factor, also could be crucial in control of pathologic angiogenesis [25], possibilities well supported by our previously reported findings. We have reported that specific histone deacetylase inhibition may suppress the VEGF gene in an indirect manner by downregulating HIF-1α with increasing p53 and von Hippel-Lindau levels in hypoxic conditions [26], a process that was effectively applied to retinal neovascularization [22]. Moreover, we found that VEGF expression was inhibited by the destabilization of HIF-1α protein via significantly reduced binding of heat shock protein 90 [27], a process that was also applied to retinal neovascularization [21]. Because VEGFR-2 is the major signal transducer for angiogenesis processes [2], the VEGFR-2 signaling pathway represents a good target for therapeutic intervention [28]. Recently, we also suggested that decursin inhibits VEGF-mediated angiogenesis by blocking the VEGFR-2 signaling pathway [16]. Decursin, isolated from *Angelica gigas* Nakai, is a coumarin compound with various biologic activities [12-14]. The proposed molecular targets for those activities include the protein kinase C pathway [14], which is efficiently activated in VEGFR-2–mediated angiogenesis [9,10]. In our current study, we demonstrated that decursin significantly inhibited VEGF-induced proliferation, migration, and tube formation, which is related to blocking VEGFR-2 phosphorylation. Interestingly, this anti-angiogenic activity of decursin in retinal endothelial cells was effectively applied to retinal neovascularization of OIR, the mouse model of ROP.

We found that decursin had no retinal toxicity in mice at doses of up to 50 µM, five times the therapeutically effective concentration in retinal neovascularization. Moreover, decursin never induced the upregulation of GFAP in Müller cells, which appears to be an indicator of stress in the retina [29].

Decursin may be a potent anti-angiogenic agent targeting the VEGFR-2 signaling pathway that significantly inhibits retinal neovascularization without retinal toxicity. Furthermore, decursin could be applicable in various vasoproliferative retinopathies, including diabetic retinopathy, age-related macular degeneration, and ROP.
